# Estimating prevalence of Enterovirus D111 in human and non-human primate populations using cross-sectional serology

**DOI:** 10.1099/jgv.0.001915

**Published:** 2023-11-01

**Authors:** Everlyn Kamau, Mael Bessaud, Manasi Majumdar, Javier Martin, Peter Simmonds, Heli Harvala

**Affiliations:** ^1^​ Nuffield Department of Medicine, University of Oxford, Oxford, UK; ^2^​ Institut Pasteur-Unité de Biologie des Virus Entériques, Paris, France; ^3^​ WHO Collaborating Centre for Enteroviruses and Viral Vaccines, Paris, France; ^4^​ Science Research and Innovation, Medicines and Healthcare Products Regulatory Agency, South Mimms, UK; ^5^​ Microbiology Services, NHS Blood Transfusion, London, UK

## Abstract

Enteroviruses primarily affect young children with a varying severity of disease. Recent outbreaks of severe respiratory and neurological disease due to EV-D68 and EV-A71, as well as atypical hand-foot-and-mouth-disease due to CVA6, have brought to light the potency of enteroviruses to emerge as severe human pathogens. Enterovirus D111 (EV-D111) is an enteric pathogen initially detected in Central Africa in human and wildlife samples and was recently detected in environmental samples. The natural history and epidemiology of EV-D111 are poorly studied. Here, the presence of serum neutralizing antibodies to EV-D111 was estimated in human and wildlife samples from five countries. We report high prevalence of neutralizing antibodies measured against EV-D111 in human populations (range, 55–83 %), a proxy for previous infection, which indicates active virus circulation in absence of detection in clinical cases and a high number of undiagnosed infections. Notably, seroprevalence in samples from the UK varied by age and was higher in children and older adults (1–5 and >60 years old), but lower in ages 11–60. EV-D111 seroprevalence in apes and Old World monkeys was 50 % (33–66 %), which also suggests prior exposure and supports existing knowledge of enterovirus circulation in wild and captive apes and Old World monkeys. Generally, reported cases of infection likely underestimate the prevalence of infection particularly when the knowledge of community transmission is limited. Continued serologic surveillance and detection of EV-D111 in clinical and environmental samples will allow for a more robust assessment of EV-D111 epidemiology.

## Introduction

Enterovirus D111 (EV-D111) is an emerging enterovirus initially recovered from human stool samples collected in the Democratic Republic of Congo (DRC) in 2001 but misidentified as EV-D70 [1]. Ten years later, EV-D111 was detected in a stool sample of a wild chimpanzee in Cameroon [2]. Other EV-D111 strains have been isolated from human stool samples in Central African Republic and Cameroon [3, 4] and more recently, EV-D111 was detected in sewage samples in Nigeria [5], but with only eight strains reported to date. EV-D111 most likely has enteric tropism, having been detected in faeces and resistant to acid treatment [6]. EV-D111 induces strong cytopathic effects in many cell lines including L20B, RD, Hep-2c, HEK293T, LLC-MK2 and A104 [6]. Phylogenetically, EV-D111 is most closely related to EV-D70 and EV-D120 on comparison of capsid gene sequences and does not have the additional short uORF described for enteroviruses in species A, B and C [6]. Previous phylogenetic analyses of EV-D111 non-capsid genomic regions suggested that the virus evolved through intertypic recombination and presence of two separate lineages, one now circulating among NHP populations and one among humans, perhaps consistent with a recent zoonotic transmission or geographical separation of lineages [3, 5, 6].

In assessing the threat of enteroviruses to human health, it has been often proposed that enteroviruses that are rarely identified or reported in clinical samples could emerge and spread in susceptible serologically naïve populations, potentially resulting in outbreaks of severe disease with atypical manifestations [7]. Based on this, enteroviruses with scope to cause outbreaks of severe disease might include EV-A76, A77, A81, A120, B80, B73, B97, B93, C99, C105 and D94 strains, all of which have been isolated from acute flaccid myelitis cases in African countries and/or the USA [1, 3, 8, 9], but have been infrequently reported in Western countries. Other novel and clinically rare enteroviruses including EV-C104, C105, C109, C117 and C118 were detected in samples taken from patients with mild to severe respiratory illnesses [1, 10–13]. Therefore, it is important to investigate the circulation and extent of exposure of rare enteroviruses, especially those within species D as exemplified by EV-D70 which emerged to cause an acute haemorrhagic conjunctivitis pandemic in the early 1970s.

Enterovirus infections in non-human primates (NHPs) have been reported widely [14–16] and zoonotic enterovirus transmission was reported in respiratory disease outbreaks occurring simultaneously among gorilla populations and humans residing in nearby research camps in the Central African Republic [17]. A recent study reported genetic identity of enterovirus strains obtained from wild and captive great apes and surrounding human communities [18]. EV-A76, A89, A119 and B107 strains were isolated in humans and animals in Central Africa in areas deemed to have high contacts between humans and animals, and contained genomic components derived from human and animal parental strains [2, 3, 16, 19–21]. Identification of human EV-B107 and EV-B90 strains in NHPs in Gabon was linked to cross-species transfer between humans and animals attributed to increased human-animal contact [16]. Furthermore, human populations in the West and Central African countries are generally assumed to have increased human-animal contacts due to activities such as bushmeat hunting [21–23], and this represents a bigger risk factor for cross-species enterovirus transmission [16]. This highlights potential for cross-species enterovirus transmission and importance of screening multiple hosts for enterovirus diversity.

In this study, human sera from five countries and plasma samples from captive NHPs were tested for anti-EV-D111 neutralizing antibodies to estimate the extent of exposure and prevalence of EV-D111.

## Methods

### Serum samples

Samples used for EV-D111 serology assays had been collected from human populations in Cameroon (*n*=100), the Democratic Republic of Congo (DRC, *n*=100), South Africa (*n*=100), Burkina Faso (*n*=100) and England, UK (*n*=227) (Table 1). The DRC and Cameroon were selected because EV-D111 had previously been detected there in humans [1, 3]. Burkina Faso was selected as it borders Cote D’Ivoire and is close to Nigeria, two countries where EV-D111 has been detected, and generally, West African countries are also considered to have high human mobility between them, which would facilitate virus transmission [24]. EV-D111 detection has not been reported in clinical samples in the UK and South Africa, or in their neighbouring countries, and hence these two countries were treated as control populations.

**Table 1.** Description of serum samples and the seroprevalence and geometric mean titre (GMT) estimates at two seropositivity thresholds

Country	Category	Mean age (range)	Collection year	No. Samples	Seroprevalence (≥1 : 16), GMT	Seroprevalence (≥1 : 32), GMT
UK	General population	35y (0–92)	2006, 2017	227	61%, 30.8	37%, 52.3
Burkina Faso	Blood donors	25y (18–56)	2007	100	58%, 58.8	40%, 105.7
Cameroon	General population	39y (35–45)	2007	100	77%, 57.0	60%, 81.6
Democratic Republic of Congo	Military population	39y (21–71)	2007	100	83%, 64.0	68%, 86.9
South Africa	Blood donors	25y (18–62)	2007	100	55%, 39.6	32%, 76.1

EV-D111 neutralizing antibodies were also tested in sera from 32 animals including chimpanzees (*P. troglodytes*), gorillas (*Gorilla gorilla gorilla*) and several Old World Monkeys. The samples had been collected in two wildlife sanctuaries in Cameroon for veterinary welfare purposes [25].

### Virus neutralization assay

The EV-D111 isolate CAF-OUP-05–059 was obtained from the European Virus Archive and had been cultured in rhabdomyosarcoma (RD) cells from stool samples of children with acute flaccid paralysis [6]. The microneutralization assay was as previously described [26, 27]. Briefly, serum was inactivated for 30 min at 56 °C, and then diluted two-fold serially from 1 : 8 to 1 : 1024. From this, 50 µl of diluted sera and 50 µl of 100 TCID_50_ of virus stock were mixed in 96-well microplates and incubated at 37 °C for 1 h. Then, 100 µl of RD cell suspension (containing 20000 cells) was added and checked for cytopathic effect (CPE) after 4–5 days of incubation at 37 °C, in 5 % CO_2_. Each sample was tested in duplicate. There is no commercially available standard serum EV-D111 and instead, pooled adult sera that inhibited virus replication at a dilution >1 : 32 were used as positive controls. Inactivated horse serum (ATCC) was used as negative control. A virus back-titration assay and a cell control (‘no virus’ control) were included for each batch of tests. Neutralizing antibody titres were reported as the reciprocal of the highest serum dilution that inhibited 50 % virus growth in both replicates.

### Statistical analyses

Fisher’s exact tests were used to determine associations between seroprevalence to EV-D111 and that of other enteroviruses (EV-D68, EV-A71, CVA6 and poliovirus types 1, 2 and 3. To determine associations between antibody levels to the different enteroviruses, neutralizing antibody titres were compared by Spearman’s rank correlation test on paired data. Statistical analyses were computed in R version 4.2.1 (https://www.r-project.org) and by SPSS version 27.

## Results

### Measurement of EV-D111 seroprevalence

In this study, the prevalence of antibodies against EV-D111 in different human populations was assessed using a live virus neutralization assay. Serial serum dilutions were incubated with EV-D111, and endpoints determined by visual inspection for virus cytopathic effect (CPE) after 3 days. Test samples showed a range of neutralization titres (Fig. S1, available in the online version of this article; Supplementary Materials) ranging from <1 : 8 to 1 : 1024. However, there was no obvious cutoff threshold titre for assigning samples as seropositive or seronegative; for study purposes we have therefore described seroprevalence at two chosen thresholds, where seropositivity was assigned to samples with neutralizing antibody titres of ≥1 : 16 and ≥1 : 32.

### Specificity of EV-D111 neutralizing antibody detection

To address the possibility that neutralizing antibodies detected to EV-D111 may not be fully serotype-specific, we compared seropositivity and titres of EV-D111 neutralizing antibody to those previously determined against specific strains of EV-D68, EV-A71 and CVA6 for the UK samples (see [26, 27] for further details on the assays and virus strains; and Fig. S2), and against poliovirus types 1, 2 and 3 (see Supplementary Information for details on the poliovirus assays and strains). In a pairwise manner, we checked for significant association or independence between EV-D111 serostatus assignments (seropositive or seronegative) and that of the heterologous enteroviruses and poliovirus types (Table 2, Fishers exact tests). At the antibody titre cutoff of <1 : 16, 33 % (75/227) of the tested samples were EV-D111 seronegative. Of these, 95 % (*n*=71) were EV-D68 seropositive, 91 % (*n*=68) were CVA6 seropositive and 81 % (*n*=61) were EV-A71 seropositive. Additionally, 30 % (*n*=32) EV-D111 seronegative samples were seropositive to all three poliovirus types combined. Overall, at the titre cutoff of ≥1 : 16, there was no statistically significant association between seroprevalence of EV-D111 and the other enterovirus serotypes (*p*-value^a^ >0.05, Table 2).

**Table 2.** Assessment of EV-D111 neutralizing antibody specificity as described below*. The numbers in the first two rows of the table indicate the number of samples in the respective comparisons between enteroviruses. Antibody testing against EV-D68, CVA6, EV-A71 and polioviruses (PV) had been done against specific strains of each of these viruses (see Supplementary Information)

	EV-D68 (−)	EV-D68 (+)	CVA6(−)	CVA6(+)	EV-A71 (−)	EV-A71 (+)	PV1 (−)	PV1 (+)	PV2 (−)	PV2 (+)	PV3 (−)	PV3 (+)
**EV-D111 (−**)	4	71	7	68	14	61	1	36	0	37	5	32
**EV-D111 (+**)	8	144	28	124	30	122	4	63	0	67	10	57
***p*-value^a^**	1		0.08		1		0.65		1		1	
**cor.coeff^b^**	0.013		0.124		0.104		0.09		0.1		0.16	
***p*-value^b^**	0.851		0.118		0.063		0.36		0.3		0.09	

*assessment was done by pairwise comparison between heterologous enteroviruses.

p-value^a^ are derived from Fisher’s exact tests (two-sided) comparing seroprevalence among the different serotypes.

cor.coeff^b^ show Spearman rank correlation coefficients and p-value^b^ show significance at the 0.05 level (two-tailed) from antibody titre comparisons between the different serotypes. (−) indicates seronegative result (no detectable neutralizing antibodies) and (+) indicates seropositive result (detectable neutralizing antibodies).

**Fig. 1.**
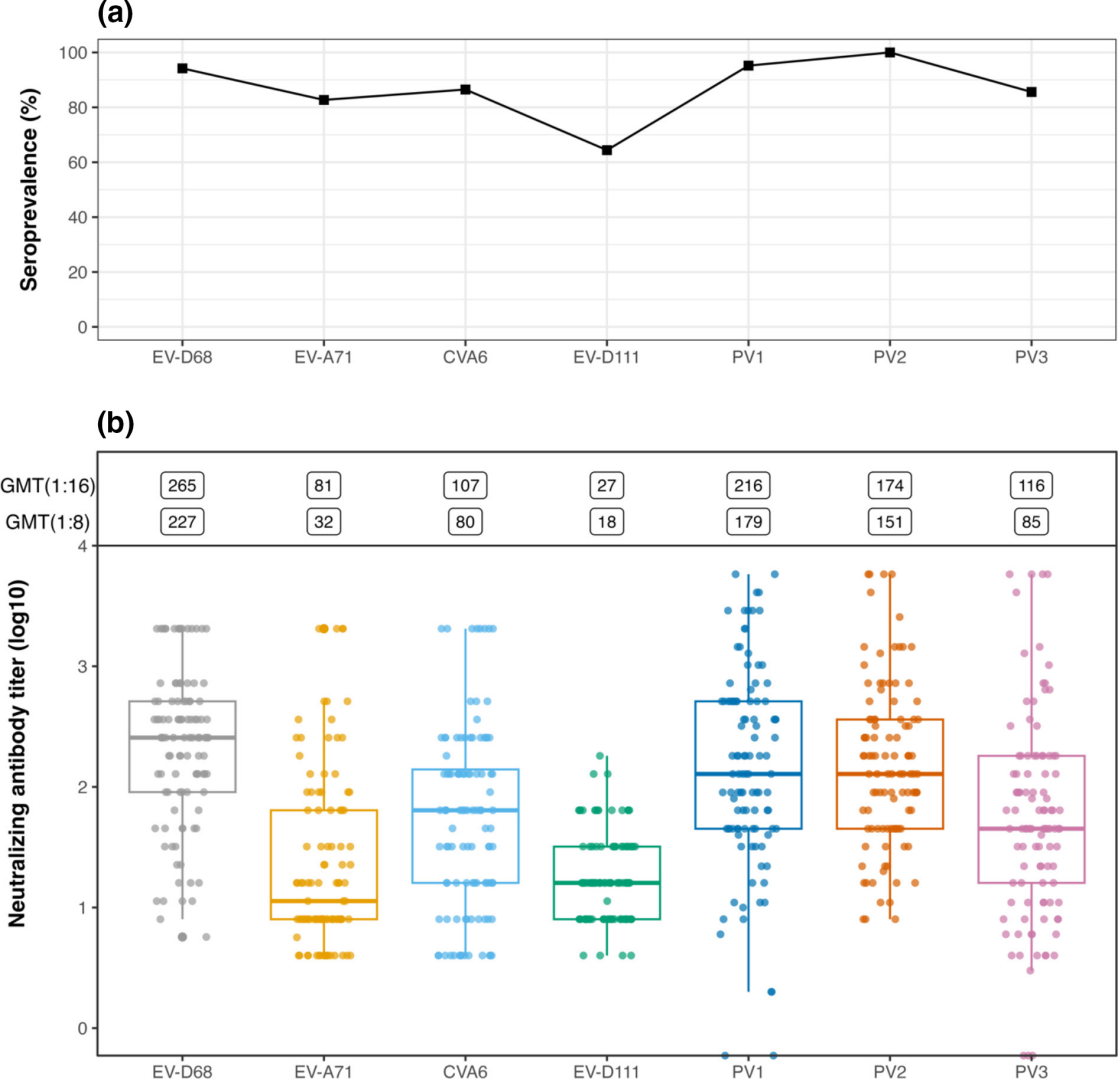
Distribution of EV-D68, EV-A71, CVA6, EV-D111 and poliovirus neutralizing antibodies in the UK samples tested in this study (*n*=104). Seroprevalence data are shown in panel A and geometric mean titres and neutralizing antibody titres are shown in panel B; and indicated in Table S2. PV1 – poliovirus type 1, PV2 – poliovirus type 2, PV3 – poliovirus type 3.

At the antibody titre cutoff of ≥1 : 32, 63 % (143/227) of the tested samples were EV-D111 seronegative, for which 96 % were EV-D68 seropositive, 87 % were CVA6 seropositive, 80 % were EV-A71 seropositive and 88 % were seropositive to all three poliovirus types combined. Similarly at the titre cutoff of ≥1 : 32, there was no statistically significant association between seroprevalence of EV-D111 and the other viruses (*p*-value>0.05, Fishers exact tests). There were no associations between neutralizing antibody titre levels to EV-D111 with those of heterologous enteroviruses by non-parametric Spearman rank correlation tests. Other comparisons and parallel analyses of antibody specificity among the enteroviruses are described in the Supplementary Information (Table S1). Overall, we obtained no evidence for neutralizing antibody cross-reactivity between the serotypes tested. Antibody titres to the four enteroviruses and the three polioviruses studied here are depicted in Fig. 1. Geometric mean titres and seropositivity across all viruses are shown in Fig. 1 and in Table S2.

### Seroprevalence of EV-D111 neutralizing antibodies in different human population groups

**Fig. 2.**
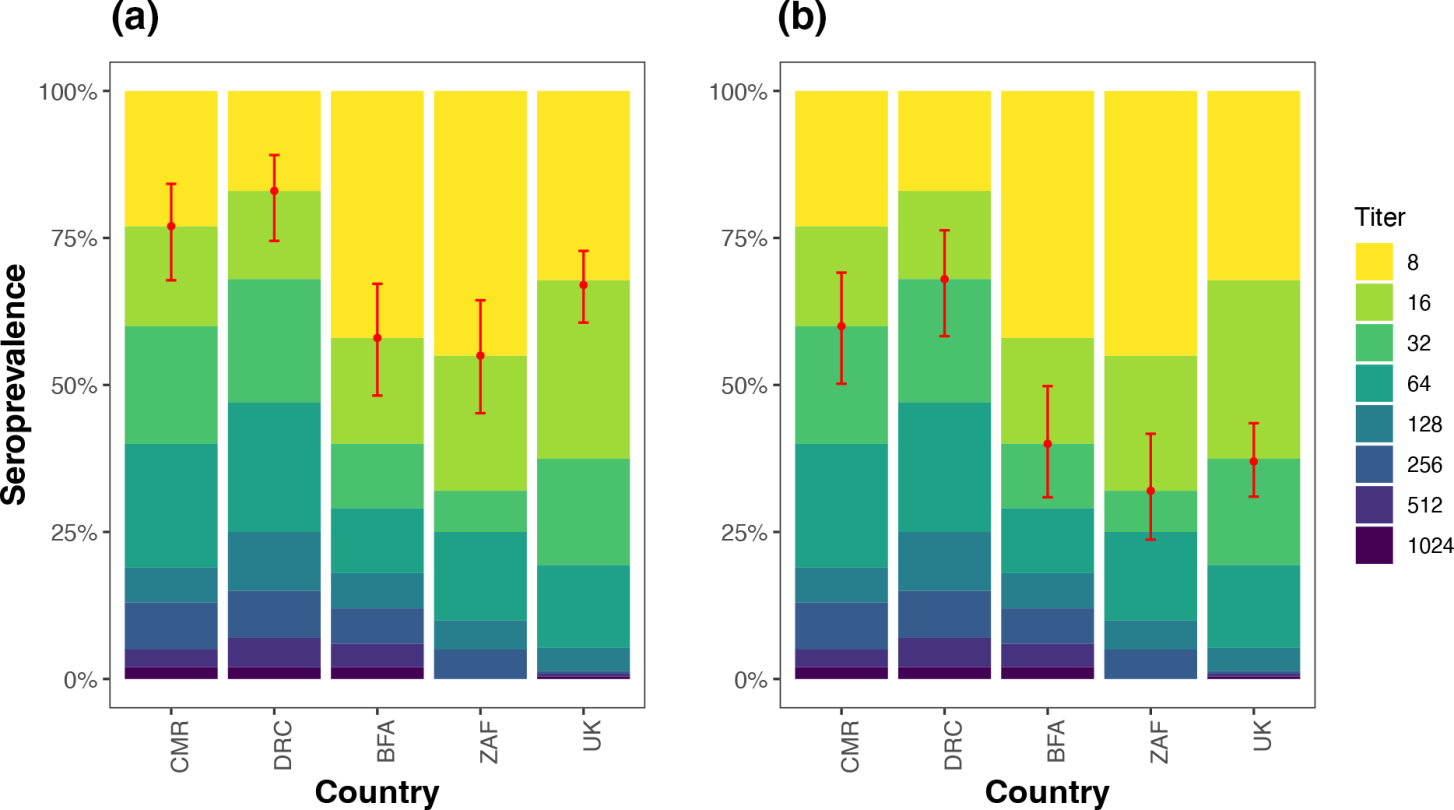
Seroprevalence of EV-D111 in representative samples from five countries. The coloured bars show the percentage of samples with neutralizing antibody titres 1 : 8 to ≥1 : 1024. Point estimates of the percentage that were seropositive at ≥1 : 16 (**a**) and ≥1 : 32 (**b**) endpoint titres are shown in red and error bars indicate 95 % binomial confidence intervals. (Abbreviations: CMR – Cameroon, DRC – Democratic Republic of Congo, BFA – Burkina Faso, ZAF – South Africa, UK – United Kingdom).

The proportion of seropositive samples with antibody titres of ≥1 : 16 ranged from 55–83% and was highest in the DRC and lowest in South Africa. The proportion of seropositive samples with antibody titres of ≥1 : 32 ranged from 32 % in South Africa to 68 % in the DRC (Table 1). Generally, samples from the DRC had higher antibody levels followed by the Cameroonian samples, twice as high as antibody levels in samples from South Africa and UK (Fig. 2).

**Fig. 3.**
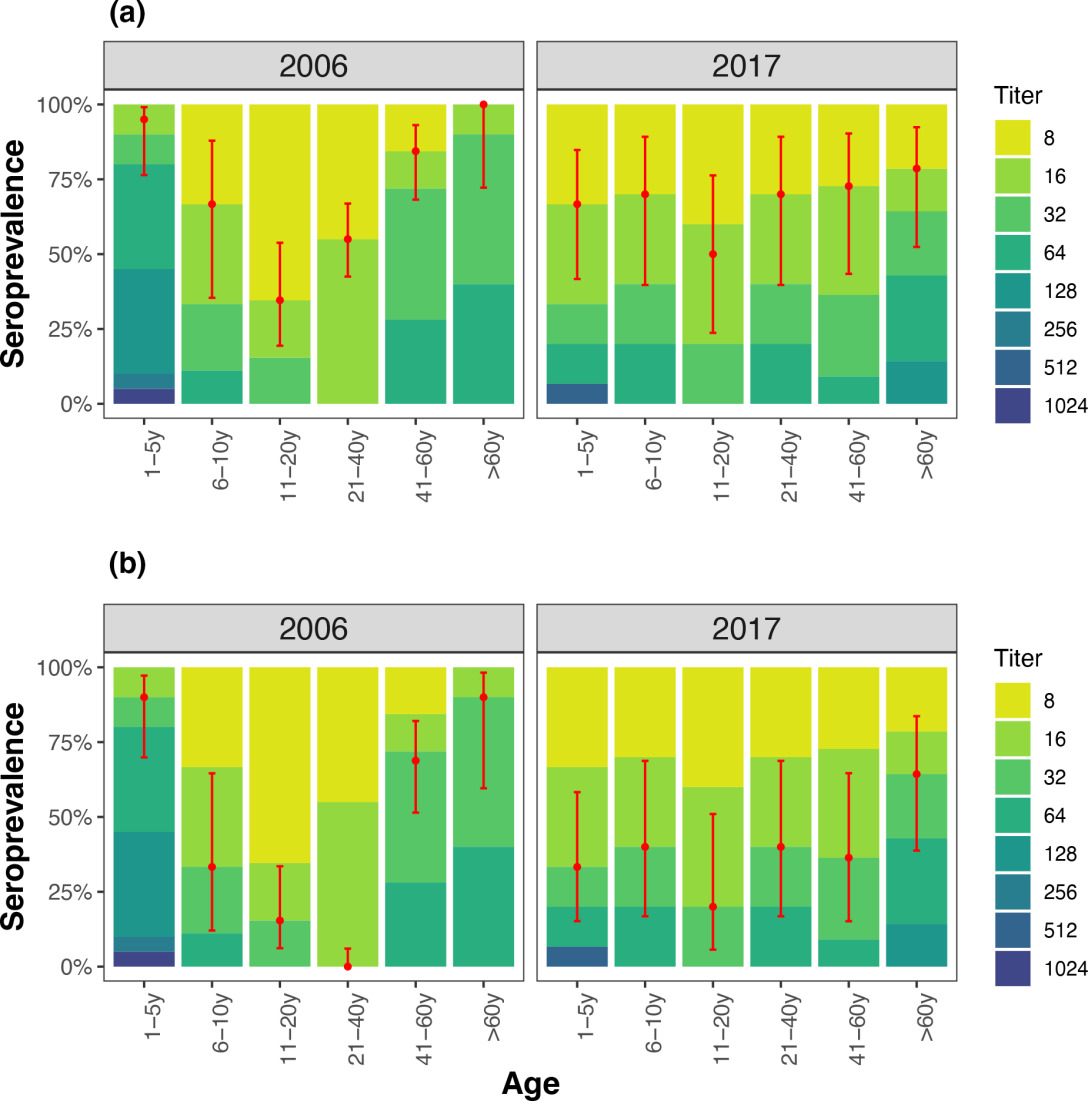
Seroprevalence of EV-D111 in representative samples from the UK. The coloured bars show the percentage of samples with neutralizing antibody titres 1 : 8 to ≥1 : 1024. Point estimates of the percentage that were seropositive at ≥1 : 16 (**a**) and ≥1 : 32 (**b**) thresholds are shown in red and the error bars indicate 95 % binomial confidence intervals.

For the UK samples, seroprevalence at the titre of ≥1 : 16 ranged from 28 % in the 21–40 year-olds to 95 % in the 1–5 years-old and 100 % in adults older than 60 years in 2006, compared to 45 % in 11–20 year-olds to 80 % in over-60-year-olds in 2017 (Table 3, Fig. 3). At the titre of ≥1 : 32, all 21–40 year-olds were classed as seronegative whereas the seroprevalence remained high in the 1–5 year-olds (90%) and in adults over 69 years (81%) in 2006 but were lower in 2017 (33 and 60 %, respectively) (Table 3, Fig. 3). Consistently, EV-D111 geometric mean titres (GMTs) in the UK samples were highest in children aged 1–5 years (84 [95 % CI, 55–128] in 2006 and 34 [95 % CI, 16–76] in 2017) and in adults older than 60 years (37 [95 % CI, 27–49] in 2006 and 47 [95 % CI, 32–57] in 2017) (Table 3). Overall, antibody levels were lower in the ages between 11 and 40 years, both for samples obtained in 2006 and 2017 (Fig. 3).

**Table 3.** Seroprevalence of EV-D111 in the UK samples collected in 2006 and 2017, stratified by age groups. Results are shown for two seropositivity thresholds

	2006			2017		
**Age group**	***n***	**Seroprevalence** (**≥1 : 16), GMT**	**Seroprevalence** (**≥1 : 32), GMT**	***n***	**Seroprevalence** (**≥1 : 16), GMT**	**Seroprevalence** (**≥1 : 32), GMT**
1–5y	20	95%, 84.1	90%, 92.3	15	66.7%, 34.3	33.3%, 73.5
6–10y	9	66.7%, 25.4	33.3%, 40.3	9	77.8%, 29.0	44.4%, 45.2
11–20y	20	40%, 21.7	20%, 32.0	11	45.5%, 21.1	18.2%, 32.0
21–40y	64	28%, 16.0	0, 0	10	70%, 29.0	40%, 45.2
41–60y	33	84.8%, 36.0	66.7%, 42.5	10	70%, 24.7	40%, 38.0
>60y	11	100%, 36.7	81%, 40.3	15	80%, 46.7	60%, 59.2
**Total**	157	57.3%, 30.5	35.7%, 52.8	70	70%, 31.5	40%, 51.2

### EV-D111 seroprevalence in non-human primates

**Fig. 4.**
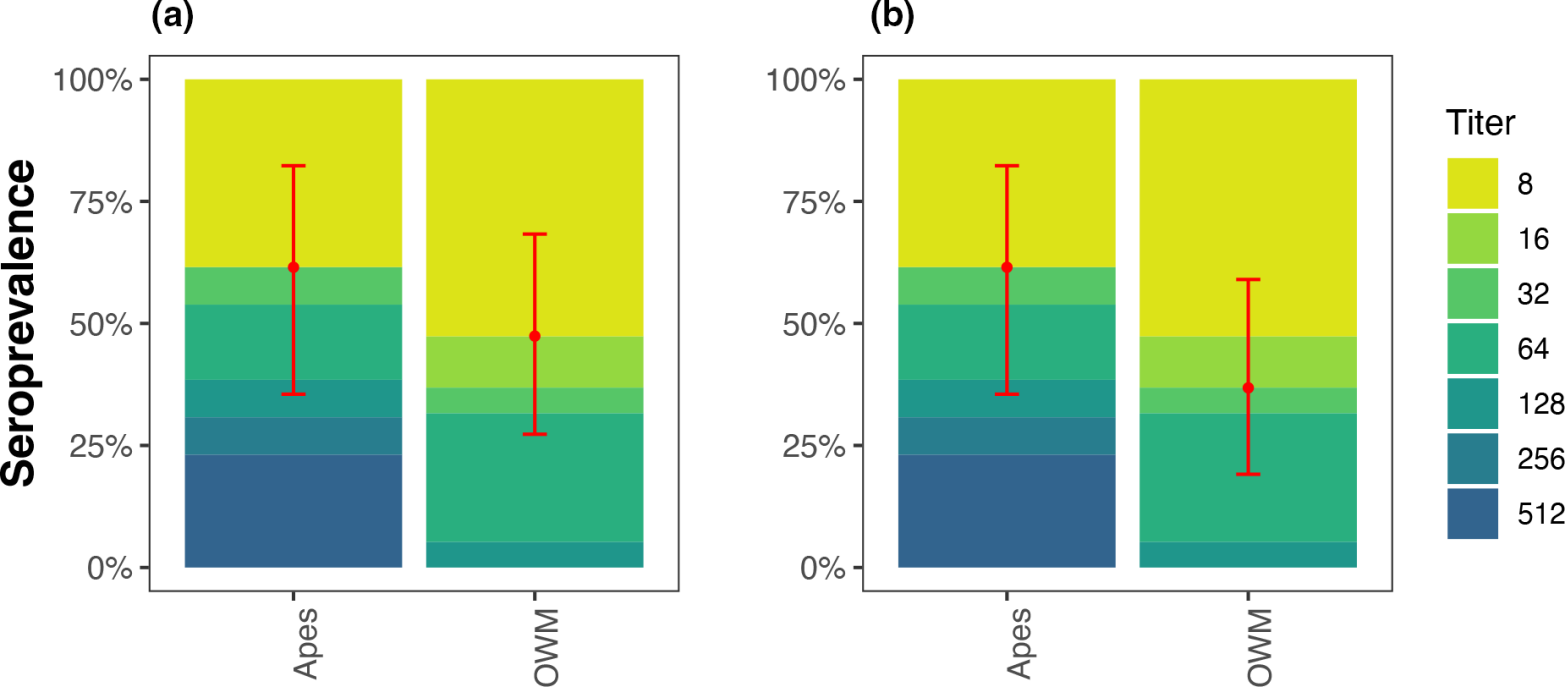
Seroprevalence of EV-D111 in representative animal sera. The coloured bars show the percentage of samples with neutralizing antibody titres 1 : 8 to ≥1 : 1024. Point estimates of the percentage that were seropositive at ≥1 : 16 (**a**) and ≥1 : 32 (**b**) endpoint titres are shown in red and error bars indicate 95 % confidence intervals of the proportions. (OWM: old world monkeys).

Anti-EV-D111 neutralizing antibodies were detected in animals in titres ranging from four to 512. Seroprevalence at the titre of ≥1 : 16 was 62 % (8/13) and 42 % (8/19) in apes and Old World Monkeys (OWM), respectively (Table 4). Among the OWMs, the highest levels of antibodies were detected in drills (*M. leucophaeus*) (75%, Table 4). At the titre of ≥32, seroprevalence was 40 % (13/32) and higher in gorillas compared to other NHP species (Table 4). Generally, gorillas and chimpanzees showed extensive previous exposure to EV-D111 (Fig. 4), demonstrating wide circulation among apes. Overall, the GMTs at antibody cutoffs of ≥1 : 16 and ≥1 : 32 were 72.0 and 57.5, respectively.

**Table 4.** Seroprevalence of EV-D111 in apes and old world monkeys

		EV-D111	
**Taxonomic name (common name**)	**No**.	**Seroprevalence (≥1 : 16**)	**Seroprevalence (≥1 : 32**)
**Apes**			
*Pan troglodytes* (chimpanzee)	8	4 (50 %)	3 (38 %)
*Gorilla gorilla gorilla* (gorilla)	5	4 (80 %)	4 (80 %)
Total	13	8 (62 %)	7 (54 %)
**Old World Monkeys**			
*Mandrillus Sphinx* (mandrill)	3	1 (33 %)	1 (33 %)
*Papio Anubis* (olive baboon)	2	1 (50 %)	1 (50 %)
*Cercopithecus preussi* (Preussi’s monkey)	2	1 (50 %)	1 (50 %)
*Cercopithecus pogonias* (crowned monkey)	2	1 (50 %)	0
*Cercopithecus mona* (mona monkey)	2	1 (50 %)	1 (50 %)
*Cercopithecus nictitans* (spot-nosed monkey)	1	0	0
*Cercopithecus pogonias* (crowned monkey)	1	0	0
*Erythrocebus patas* (patas monkey)	2	0	0
*Mandrillus leucophaeus* (drill)	4	3 (75 %)	2 (50 %)
Total	19	8 (42 %)	6 (32 %)

## Discussion

EV-D111 remains poorly understood as there is limited data on clinical disease and there are no published studies on its seroprevalence. We examined the seroprevalence of EV-D111 in human samples obtained from five countries and in serum from non-human primates. The neutralization assay used here was comparable to those used previously for seroepidemiology investigations of other enteroviruses by us [26, 27] or in other studies. However, we did not have a concrete basis for selecting a threshold or cutoff for estimating seropositivity as the titre distribution in Fig. 1 did not show a visual inflection point that could be used as a cutoff value. There are no previous studies on EV-D111 seroepidemiology that recommend an optimal cutoff, define the protective antibody titre levels, or inform on immunological background of the population. Furthermore, international standards for EV-D111 serological surveys are lacking. Determining appropriate cut-offs for seropositivity can be challenging and could hinder comparability of epidemiological data between settings and over time.

We identified highest EV-D111 seroprevalence in the Central African countries (Democratic Republic of Congo, Burkina Faso and Cameroon), where the virus was previously detected in human and animal faecal samples [2, 3]. Uncontrolled ‘bushmeat’ hunting and close interaction between humans and NHPs have been reported in the region, creating a risk of exposure to viruses circulating in wildlife and vice versa [28]. Although the sample sizes were too small to make conclusive inferences (*n*=100 per country), detection of EV-D111-specific neutralizing antibodies provides evidence for widespread asymptomatic or pauci-symptomatic infections, and endemic circulation of EV-D111. The method provides a valuable indicator of virus activity in regions of limited enterovirus surveillance. Recently, EV-D111 strains were detected in sewage in Nigeria and in faecal specimens in Angola, further supporting EV-D111 circulation in the wider African region [5, 29].

EV-D111 seroprevalence in the UK was similarly high; this was unexpected as the virus has not been detected in clinical cases or in more general population-based screening, such as sewage surveillance [30]. There are several potential reasons for these observations. Firstly, the high seroprevalence could be attributed to assay non-specific cross-reactivity with other enteroviruses. However, we demonstrated that EV-D111 neutralizing antibody titres were independent of those against EV-D68, EV-A71, CVA6 and polioviruses (Table 2). Lack of serological cross-reactivity between enterovirus serotypes has been a general principal underlying past seroepidemiology studies, and originates from marked sequence heterogeneity and structural differences between serotypes in their encoded VP1 and VP3 proteins, the target for serum neutralizing antibodies [31]. Nonetheless, some degree of antigenic relationship among enteroviruses cannot be fully ruled out given the sheer number of enteroviruses and potential for shared neutralizing epitopes (currently >100 serotypes assigned). Indeed, most serotypes have been assigned on the basis of >25 % genetic divergence in VP1 [32] rather than using the original assignment criterion of a demonstrated lack of serological cross-reactivity with other serotypes, so this possibility has not been systematically evaluated [33]. Indeed, there are reports of cross-neutralization among enteroviruses within species A although infrequent or occurring in only a small proportion of patients [34–36]. It is important to note that although cross-binding of poliovirus to the polyclonal sera obtained from EV-D111 infected mice was proposed, no data on cross-neutralization was shown to support that observation. Particularly for samples with high neutralizing antibody titres (≥1 : 32) against EV-D111 as shown here, we therefore do not believe that cross-reactivity accounts for high seroprevalences observed in the current study.

For the UK samples, we observed differences in age groups between the 2006 and 2017 timepoints. The 1–5 year age group in 2006 had a higher seroprevalence compared to the 6–10 year and 11–20 year age groups in 2017 (Table 3). Although the sample sizes were too small to make conclusive inferences, this disparity in seroprevalence could indicate lower infection rates or incidence of EV-D111 in the last decade, or waning immunity due to previous infection or vaccination.

If the high seroprevalence observed in the study reflects extensive circulation of EV-D111 in the UK and elsewhere, it may have remained undetected because of its possibly largely mild or asymptomatic nature of its infections. EV-D111 infections in the UK may also have been largely undiagnosed – non-polio enterovirus infections are not notifiable in the UK, and even if diagnosed, only samples obtained from more severe cases are forwarded to the national reference laboratory for typing [37, 38]. As discussed elsewhere [39], current monitoring of EV infections in the UK and most developed countries is not systematic. Consequently, EV infections are massively underdiagnosed, making it possible that EV-D111 and perhaps a range of other EVs have circulated undetected for many decades.

However, if EV-D111 was actively circulating in the UK, even if asymptomatically, EV-D111 strains should have been detected in sewage samples collected from the UK. A recent study using a PCR-based metagenomic approach demonstrated extensive diversity of EVs, but not EV-D111 [30], contrasting with the finding of EV-D111 strains Nigeria [5]. Since the virus has consistently been found or detected in stool samples from cases [6], its presumed enteric tropism might be expected to lead to its representation in sewage if it was circulating in the UK. However, samples from UK sewage surveillance are primarily analysed for the presence of polioviruses; and the use of metagenomic sequencing that would reveal the presence of EV-D111 had only been performed on a very small number of samples over a short time span (2015–2017) [30]. The available sampling would therefore likely be insufficient to capture short-term outbreaks of EV-D111, few and far between yet able to infect a substantial proportion of the population during its circulation.

Notwithstanding the evidence for substantial circulation of EV-D111, our findings indicate a notable residual population without EV-D111 neutralizing antibodies who are potentially susceptible to infection at least in four of the five countries studied. This could facilitate outbreaks and poses a risk of disease to a partly immunologically ‘naïve’ population. Our findings indeed contrast with the results of seroepidemiology studies for other enteroviruses, particularly in the UK, that typically report nearly 100 % seroprevalence in the adult population [26, 27].

EV-D111 seroprevalence in NHPs was slightly lower than that in human populations. Antibody cross-reactivity among different enteroviruses is feasible, and although not evaluated in the animal sera studied here and even without EV-D111 RNA detection, the seroprevalence results indicate possible prior exposure to EV-D111 and support existing knowledge of enterovirus circulation in wild and captive animals. Animals are thought to play a role in the natural cycle of enterovirus circulation and a large genetic diversity of enteroviruses either distinct to animal populations or resembling human enterovirus strains has been documented, comprising members of enterovirus species A, B, C, D, J, and H [2, 3, 16, 20, 40–44]. For instance, EV-C99, a human enterovirus, was potentially responsible for acute flaccid myelitis illness in a chimpanzee in Congo [45]. Enterovirus infections in apes are widespread and active infections were reported among mandrills and other Old World monkey species inhabiting remote regions of Cameroon without human contact [46]. Long-term faecal shedding of enteroviruses, the environmental stability of enteroviruses and contamination of nest sites, could perpetuate infections among NHPs [25]. Higher population connectivity among animal groups and supply of susceptible animals may support cross-species transmission and indigenous enterovirus circulation in the animal population [47]. Novel enterovirus types, including EV-B112 in a chimpanzee, EV-B113 in a mandrill, EV-122 and EV-123 in wild monkeys, were identified more recently suggesting that NHPs could be potential sources of new enterovirus infections in humans [16, 19]. Detection of EV-D111 neutralizing antibodies in wild and captive animals could indicate their role as reservoirs and possible sources of virus infection or emergence. Given their close evolutionary relationship with humans it is therefore essential to continue exploring and monitoring EV circulation in NHPs.

This is the first seroepidemiology study of EV-D111 where we provide substantial serology-based evidence for EV-D111 circulation in humans and animals, and a further indication of its wide host range. Further serology studies are needed to understand the ecology and epidemiology of EV-D111. The correlates of immunity of EV-D111 are unknown and considering the relatively low to moderate seroprevalence reported here for adult UK population aged between 20 and 40 years, and its detection in acute flaccid paralysis cases, EV-D111 may emerge in immunologically naïve human populations and should therefore be considered a potential public health risk.

## Supplementary Data

Supplementary material 1

## References

[1] Junttila N, Lévêque N, Kabue JP, Cartet G, Mushiya F (2007). New enteroviruses, EV-93 and EV-94, associated with acute flaccid paralysis in the democratic Republic of the Congo. J Med Virol.

[2] Harvala H, Sharp CP, Ngole EM, Delaporte E, Peeters M (2011). Detection and genetic characterization of enteroviruses circulating among wild populations of chimpanzees in Cameroon: relationship with human and simian enteroviruses. J Virol.

[3] Sadeuh-Mba SA, Bessaud M, Massenet D, Joffret M-L, Endegue M-C (2013). High frequency and diversity of species C enteroviruses in Cameroon and neighboring countries. J Clin Microbiol.

[4] Bessaud M, Pillet S, Ibrahim W, Joffret M-L, Pozzetto B (2012). Molecular characterization of human enteroviruses in the Central African Republic: uncovering wide diversity and identification of a new human enterovirus A71 genogroup. J Clin Microbiol.

[5] Majumdar M, Klapsa D, Wilton T, Bujaki E, Fernandez-Garcia MD (2021). High diversity of human non-polio enterovirus serotypes identified in contaminated water in Nigeria. Viruses.

[6] Sadeuh-Mba SA, Joffret M-L, Mazitchi A, Endegue-Zanga M-C, Njouom R (2019). Genetic and phenotypic characterization of recently discovered enterovirus D type 111. PLoS Negl Trop Dis.

[7] Nguyen-Tran H, Park SW, Messacar K, Dominguez SR, Vogt MR (2022). Enterovirus D68: a test case for the use of immunological surveillance to develop tools to mitigate the pandemic potential of emerging pathogens. Lancet Microbe.

[8] Fernandez-Garcia MD, Kebe O, Fall AD, Ndiaye K (2017). Identification and molecular characterization of non-polio enteroviruses from children with acute flaccid paralysis in West Africa, 2013-2014. Sci Rep.

[9] Faleye TOC, Adewumi MO, Japhet MO, David OM, Oluyege AO (2017). Non-polio enteroviruses in faeces of children diagnosed with acute flaccid paralysis in Nigeria. Virol J.

[10] Barnadas C, Midgley SE, Skov MN, Jensen L, Poulsen MW (2017). An enhanced enterovirus surveillance system allows identification and characterization of rare and emerging respiratory enteroviruses in Denmark, 2015-16. J Clin Virol.

[11] Tokarz R, Hirschberg DL, Sameroff S, Haq S, Luna G (2013). Genomic analysis of two novel human enterovirus C genotypes found in respiratory samples from Peru. J Gen Virol.

[12] Piralla A, Daleno C, Girello A, Esposito S, Baldanti F (2015). Circulation of two enterovirus C105 (EV-C105) lineages in Europe and Africa. J Gen Virol.

[13] Horner LM, Poulter MD, Brenton JN, Turner RB (2015). Acute flaccid paralysis associated with novel enterovirus C105. Emerg Infect Dis.

[14] Fieldhouse JK, Wang X, Mallinson KA, Tsao RW, Gray GC (2018). A systematic review of evidence that enteroviruses may be zoonotic. Emerg Microbes Infect.

[15] Nix WA, Jiang B, Maher K, Strobert E, Oberste MS (2008). Identification of enteroviruses in naturally infected captive primates. J Clin Microbiol.

[16] Mombo IM, Lukashev AN, Bleicker T, Brünink S, Berthet N (2017). African non-human primates host diverse enteroviruses. PLoS One.

[17] Grützmacher KS, Köndgen S, Keil V, Todd A, Feistner A (2016). Codetection of respiratory syncytial virus in habituated Wild Western Lowland gorillas and humans during a respiratory disease outbreak. Ecohealth.

[18] Amona I, Medkour H, Akiana J, Davoust B, Tall ML (2020). Enteroviruses from humans and great apes in the Republic of Congo: recombination within enterovirus C serotypes. Microorganisms.

[19] Sadeuh-Mba SA, Bessaud M, Joffret M-L, Endegue Zanga M-C, Balanant J (2014). Characterization of enteroviruses from non-human primates in Cameroon revealed virus types widespread in humans along with candidate new types and species. PLoS Negl Trop Dis.

[20] Harvala H, Van Nguyen D, McIntyre C, Ahuka-Mundeke S, Ngole EM (2014). Co-circulation of enteroviruses between apes and humans. J Gen Virol.

[21] Mossoun A, Pauly M, Akoua-Koffi C, Couacy-Hymann E, Leendertz SAJ (2015). Contact to non-human primates and risk factors for zoonotic disease emergence in the Taï Region, Côte d’Ivoire. Ecohealth.

[22] Mossoun A, Calvignac-Spencer S, Anoh AE, Pauly MS, Driscoll DA (2017). Bushmeat hunting and zoonotic transmission of simian T-lymphotropic virus 1 in Tropical West and Central Africa. J Virol.

[23] Paige SB, Frost SDW, Gibson MA, Jones JH, Shankar A (2014). Beyond bushmeat: animal contact, injury, and zoonotic disease risk in Western Uganda. Ecohealth.

[24] Omoleke SA, Mohammed I, Saidu Y (2016). Ebola viral disease in West Africa: a threat to global health, economy and political stability. J Public Health Afr.

[25] Harvala H, McIntyre CL, Imai N, Clasper L, Djoko CF (2012). High seroprevalence of enterovirus infections in apes and old world monkeys. Emerg Infect Dis.

[26] Kamau E, Harvala H, Blomqvist S, Nguyen D, Horby P (2019). Increase in enterovirus D68 infections in young children, United Kingdom, 2006-2016. Emerg Infect Dis.

[27] Kamau E, Nguyen D, Celma C, Blomqvist S, Horby P (2021). Seroprevalence and virologic surveillance of enterovirus 71 and coxsackievirus A6, United Kingdom, 2006-2017. Emerg Infect Dis.

[28] Wolfe ND, Daszak P, Kilpatrick AM, Burke DS (2005). Bushmeat hunting, deforestation, and prediction of zoonoses emergence. Emerg Infect Dis.

[29] Chern S-WW, Gumede N, Castro CJ, Nix WA, Ng TFF (2021). Whole-genomesequences of enteroviruses D94 and D111 isolated from stool specimens in Angola. Microbiol Resour Announc.

[30] Majumdar M, Sharif S, Klapsa D, Wilton T, Alam MM (2018). Environmental surveillance reveals complex enterovirus circulation patterns in human populations. Open Forum Infect Dis.

[31] Zhang W, Dai W, Zhang C, Zhou Y, Xiong P (2018). A virus-like particle-based tetravalent vaccine for hand, foot, and mouth disease elicits broad and balanced protective immunity. Emerg Microbes Infect.

[32] Oberste MS, Maher K, Kilpatrick DR, Pallansch MA (1999). Molecular evolution of the human enteroviruses: correlation of serotype with VP1 sequence and application to picornavirus classification. J Virol.

[33] Lugo D, Krogstad P (2016). Enteroviruses in the early 21st century: new manifestations and challenges. Curr Opin Pediatr.

[34] Takahashi S, Metcalf CJE, Arima Y, Fujimoto T, Shimizu H (2018). Epidemic dynamics, interactions and predictability of enteroviruses associated with hand, foot and mouth disease in Japan. J R Soc Interface.

[35] Lin Y, Wen K, Pan Y, Wang Y, Che X (2011). Cross-reactivity of anti-EV71 IgM and neutralizing antibody in series sera of patients infected with enterovirus 71 and coxsackievirus A 16. J Immunoassay Immunochem.

[36] Nguyet LA, Thanh TT, Nhan LNT, Hong NTT, Nhu LNT (2020). Neutralizing antibodies against enteroviruses in patients with hand, foot and mouth disease. Emerg Infect Dis.

[37] Majumdar M, Martin J (2018). Detection by direct next generation sequencing analysis of emerging enterovirus D68 and C109 strains in an environmental sample from Scotland. Front Microbiol.

[38] Kadambari S, Bukasa A, Okike IO, Pebody R, Brown D (2014). Enterovirus infections in England and Wales, 2000-2011: the impact of increased molecular diagnostics. Clin Microbiol Infect.

[39] Harvala H, Benschop KSM, Berginc N, Midgley S, Wolthers K (2021). European non-polio enterovirus network: introduction of hospital-based surveillance network to understand the true disease burden of non-polio enterovirus and parechovirus infections in Europe. Microorganisms.

[40] Oberste MS, Maher K, Michele SM, Belliot G, Uddin M (2005). Enteroviruses 76, 89, 90 and 91 represent a novel group within the species human enterovirus A. J Gen Virol.

[41] Nielsen SCA, Mourier T, Baandrup U, Søland TM, Bertelsen MF (2012). Probable transmission of coxsackie B3 virus from human to chimpanzee, Denmark. Emerg Infect Dis.

[42] Miyagi J, Tsuhako K, Kinjo T, Iwamasa T, Kamada Y (1999). Coxsackievirus B4 myocarditis in an orangutan. Vet Pathol.

[43] Oberste MS, Feeroz MM, Maher K, Nix WA, Engel GA (2013). Naturally acquired picornavirus infections in primates at the Dhaka Zoo. J Virol.

[44] Oberste MS, Feeroz MM, Maher K, Nix WA, Engel GA (2013). Characterizing the picornavirus landscape among synanthropic nonhuman primates in Bangladesh, 2007 to 2008. J Virol.

[45] Mombo IM, Berthet N, Lukashev AN, Bleicker T, Brünink S (2015). First detection of an enterovirus C99 in a captive chimpanzee with acute flaccid paralysis, from the tchimpounga chimpanzee rehabilitation center, Republic of Congo. PLoS One.

[46] Van Nguyen D, Harvala H, Ngole EM, Delaporte E, Woolhouse MEJ (2014). High rates of infection with novel enterovirus variants in wild populations of mandrills and other old world monkey species. J Virol.

[47] Bailes E, Gao F, Bibollet-Ruche F, Courgnaud V, Peeters M (2003). Hybrid origin of SIV in chimpanzees. Science.

[48] Wise J (2009). New initiative launched to track emergent pandemics. Lancet Infect Dis.

[49] Sharp CP, Vermeulen M, Nébié Y, Djoko CF, LeBreton M (2010). Changing epidemiology of human parvovirus 4 infection in sub-Saharan Africa. Emerg Infect Dis.

