# Supplementary material for: Estimating prevalence of Enterovirus D111 in human and non-human primate populations using cross-sectional serology
Source: J Gen Virol. 2023 Nov 1;104(11):001915. doi: 10.1099/jgv.0.001915 (PMC10768692; doi:10.1099/jgv.0.001915)

SUPPLEMENTARY INFORMATION

***Specificity of enterovirus neutralizing antibody detection**.* In this study, 227 samples were tested for neutralizing antibodies against specific contemporary strains of EV-D68, EV-A71, CVA6 and EV-D111. Specifically, EV-D68 genotype B3 and CVA6 genotype D strains circulating in 2016 and a genogroup B4 strain of EV-A71 collected in 2004 were used for testing (see [26, 27] for further details on the EV-D68, EV-A71 and CVA6 virus strains). Samples were considered EV-D68, EV-A71 and CVA6 seropositive if they had antibody titers of ≥1:16, ≥1:8 and ≥1:8, respectively.

(i) EV-D68. Of these, 5.3% (12/227) were EV-D68 seronegative (titer <1:16) for which 75% (9/12) were CVA6 seropositive, 50% (6/12) were EV-A71 seropositive and 67% (8/12) were EV-D111 seropositive.

(ii) EV-A71. Out of 227, 19.4% (44/227) were EV-A71 seronegative (titer <1:8) for which 86% (38/44) were EV-D68 seropositive, 73% (32/44) were CVA6 seropositive and 68% (30/44) were EV-D111 seropositive.

(iii) CVA6. 15.4% (35/227) samples were CVA6 seronegative (titer <1:8) for which 91% (32/35) were EV-D68 seropositive, while 66% (23/35) and 80% (28/35) were EV-A71 and EV-D111 seropositive, respectively.

There was a statistically significant correlation between EV-A71 and EV-D68 antibody titer levels ($p = 0.028$, Table S1). Antibody titers to EV-D68, EV-A71 and CVA6 in the 227 samples are depicted in Figure S2.

Detection of poliovirus neutralizing antibodies. Of the 227 samples that had been tested against EV-D68, EV-A71, CVA6 and EV-D111, 104 had enough material left and were tested for neutralizing antibodies against poliovirus (PV) serotypes 1, 2 and 3.

Serum neutralizing antibodies to the three-poliovirus serotypes were measured at the Medicines & Healthcare products Regulatory Agency (MHRA), UK, following the WHO protocol with Sabin poliovirus 1 and 3 and Type 2 S19 as challenge viruses [World Health Organization Polio laboratory manual WHO/IVB/04.10, Geneva (2004)]. The International Reference for anti-poliovirus serum (82/585) was used as a working reference and tested in parallel to confirm the

validity and sensitivity of the tests. The neutralizing antibody titers were determined as the highest dilution of the serum that inhibited each type of PV infection and samples were considered poliovirus seropositive if they had antibody titers of ≥1:8 as indicated in other studies [50].

(i) Of the 104, 37 were EV-D111 seronegative at the EV-D111 antibody titer <1:16, for which 36 were PV1 seropositive, while all 37 were PV2 seropositive and 32 were PV3 seropositive.

(ii) 71/104 were EV-D111 seronegative at the EV-D111 antibody titer <1:32, for which 69 were PV1 seropositive, all 71 were PV2 seropositive and 63 were PV3 seropositive.

(iii) 16/104 samples were seronegative to all polioviruses combined (titer <1:8 to each poliovirus type). Of these, 12/16 were EV-A71 seropositive, 15/16 were CVA6 seropositive and all 16 were EV-D68 seropositive.

There was statistically significant correlation between the poliovirus types (p <0.001, Table S1) but none between the poliovirus types and EV-A71, EV-D68 titers or CVA6 (p >0.05, Table S1). Geometric mean titers to the four enteroviruses and the three poliovirus types in the 104 samples are listed in Table S2.

Table S1 Assessment of neutralizing antibody specificity to three enteroviruses and three poliovirus (PV) types. See table footnote# for details on the analysis performed.

	**EV-D68**	**EV-A71**	**CVA6**	**PV1**	**PV2**	**PV3**
**EV-D68**						
cor.coeff	-	-	-	-	-	-
p-value	-	-	-	-	-	-
**EV-A71**						
cor.coeff	0.146	-	-	-	-	-
p-value	0.028	-	-	-	-	-
**CVA6**						
cor.coeff	0.105	0.09	-	-	-	-
p-value	0.114	0.175	-	-	-	-
**PV1**						
cor.coeff	-0.04	-0.04	0.04	-	-	-
p-value	0.64	0.67	0.67	-	-	-
**PV2**						
cor.coeff	-0.04	0.01	0.03	0.55	-	-
p-value	0.68	0.9	0.75	<0.001	-	-
**PV3**						
cor.coeff	0.004	-0.1	-0.13	0.48	0.55	-
p-value	0.97	0.29	0.18	<0.001	<0.001	-

#*assessment was done through pairwise comparison of serology data of heterologous enteroviruses. cor.coeff values show Spearman rank correlation coefficients and p-value values show significance at the 0.05 level (2-tailed) from antibody titer comparisons between the different serotypes.*

Table S2 Geometric mean titers (GMT) and seropositivity in the 104 UK samples tested against EV-D68, EV-A71, CVA6, EV-D111 and poliovirus types. GMT are calculated for seropositive samples only.

**Virus**	**GMT**	**Seropositivity**
EV-D68	(titer ≥8): 227<br>(titer ≥16): 265.2	(titer ≥16): 94.2%
EV-A71	(titer ≥8): 32.0<br>(titer ≥16): 81	(titer ≥8): 82.7%
CVA6	(titer ≥8): 80.0<br>(titer ≥16): 107	(titer ≥8): 86.5%
EV-D111	(titer ≥8): 18<br>(titer ≥16): 27.5	(titer ≥16): 64.4%
Poliovirus (PV)		
PV-1	(titer ≥8): 178.7<br>(titer ≥16): 216	(titer ≥8): 95.2%
PV-2	(titer ≥8): 151.4<br>(titer ≥16): 174	(titer ≥8): 100%
PV-3	(titer ≥8): 85.0<br>(titer ≥16): 116	(titer ≥8): 85.6%

Figure captions

Figure S1 Distribution of EV-D111 virus neutralizing antibodies in the samples tested in this study.

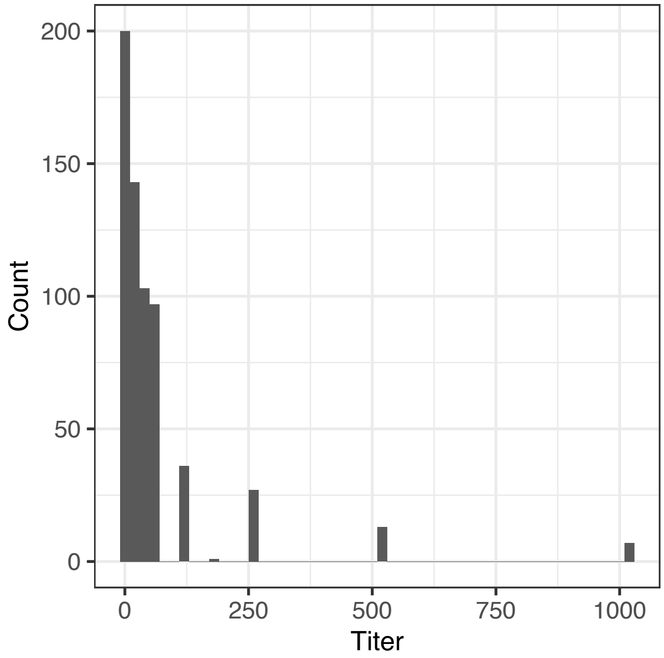

Figure S2 Distribution of EV-D68, EV-A71, CVA6 and EV-D111 neutralizing antibodies in the UK samples analyzed in this study (n=227).

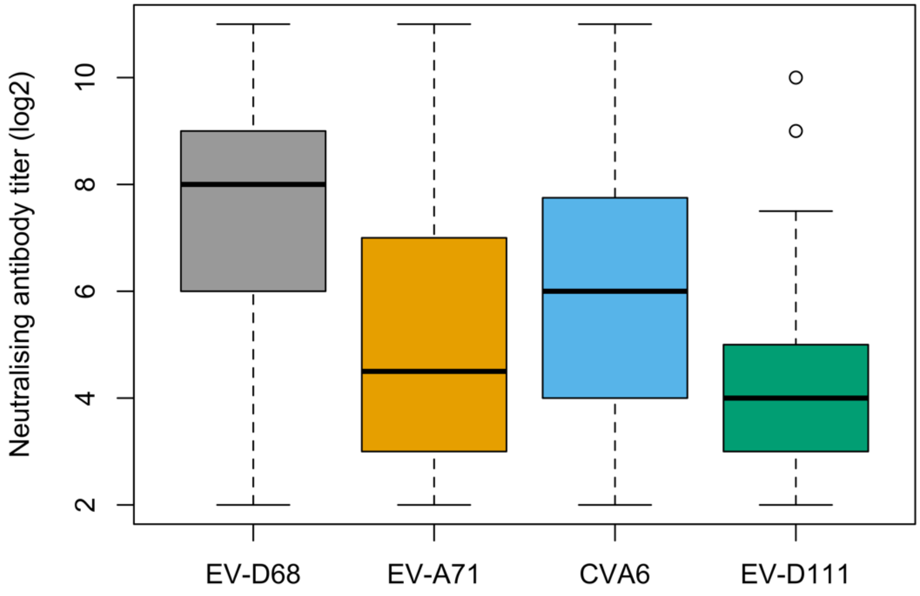

Supplement: Supplementary material 1 [file jgv-104-1915-s001.pdf]
